# Genomic analysis of PLNTY-like tumor progression into epithelioid glioblastoma: a case report

**DOI:** 10.1186/s40478-025-02209-3

**Published:** 2026-01-08

**Authors:** Sonja Mäntylä, Anssi Nurminen, Sanna Huovinen, Serafiina Jaatinen, Teppo Haapaniemi, Riikka Nurminen, Ismaïl Hermelo, Stefanie Volz, Kendra K. Maaß, Kristian W. Pajtler, Kristiina Nordfors, Hannu Haapasalo, Matti Nykter, Joonas Haapasalo, Kirsi J. Rautajoki

**Affiliations:** 1 Faculty of Medicine and Health Technology, Tampere University, Arvo Ylpön Katu 34, 33520 Tampere, Finland; 2 TAYS Cancer Center, Tampere University Hospital, Arvo Ylpön Katu 34, 33520 Tampere, Finland; 3 Fimlab Laboratories Ltd., Tampere University, Arvo Ylpön Katu 4, 33520 Tampere, Finland; 4 Hopp Children’s Cancer Center Heidelberg (KiTZ), Heidelberg, Germany; 5 Division of Pediatric Neuro Oncology, German Cancer Research Center, German Cancer Consortium (DKTK), Heidelberg, Germany; 6 Department of Pediatric Oncology, Hematology, Immunology and Pulmonology, Heidelberg University Hospital, Heidelberg, Germany; 7 Department of Pediatric Hematology and Oncology, Tampere University Hospital, Elämänaukio 2, 33520 Tampere, Finland; 8 Department of Neurosurgery, Tampere University Hospital and Tampere University, Elämänaukio 2, 33520 Tampere, Finland; 9 German Cancer Research Center (DKFZ), Im Neuenheimer Feld 280, 69120 Heidelberg, Germany

**Keywords:** Brain tumor, Cancer, Polymorphous-low-grade-neuroepithelial-of-young, Epithelioid glioblastoma, Cancer genetics

## Abstract

Polymorphous low-grade neuroepithelial tumors of the young (PLNTY) are slow- or non-progressing epileptogenic tumors, typically occurring in young adults. These tumors are highly calcified and exhibit both diffuse growth and oligodendroglioma-like patterns. Epithelioid glioblastomas (E-GB) are rare and aggressive variants of isocitrate dehydrogenase wildtype glioblastomas (GB), associated with poor overall survival. Both PLNTY and E-GB often carry oncogenic *BRAF* V600E mutation (BRAF_V600E_). In this case study, we analyzed clinical and genetic data from a single patient who, based on extensive histological and molecular evaluation, was diagnosed to carry PLNTY-like tumor that progressed into E-GB years later. The aim of our study was to uncover the genetic drivers and the evolutionary history of these tumors. Progression of the PLNTY-like tumor into E-GB was investigated using histology, chromosomal karyotyping, whole-genome sequencing (WGS), and RNA sequencing. A typical immunohistochemical stain pattern of CD34 positivity was detected in the apparent PLNTY, whereas it was depleted in the E-GB sample, as expected. WGS analysis of the PLNTY and three E-GB samples revealed four genes with shared somatic protein-altering mutations: *BRAF* (carrying BRAF_V600E_)*,* clonal in both PLNTY and E-GB, as well as *GNS, FOXRED2*, and *SSTR5*, which were subclonal in PLNTY and clonal in E-GB. Both the PLNTY-like and E-GB tumors also carried highly similar copy number alteration profiles with a prominent loss of heterozygosity (LOH) in the majority of the chromosomes, suggesting their monoclonal origin. PLNTY-like tumor was mainly diploid, and the tumor underwent a genome-wide duplication event during the progression to E-GB. Furthermore, two focal rearrangements leading to homozygous deletion of *CDKN2A/B* were detected in E-GB samples. In conclusion, this study revealed unusually extensive LOH in the histologically and genetically supported PLNTY-like tumor that progressed into E-GB. Notably, only a limited set of genetic alterations was associated with malignant transformation beyond genome duplication, the *CDKN2A/B* inactivation representing the best-known oncogenic driver for malignant transformation. These findings suggest that genomic profiling may be a valuable tool for the diagnosis and prognostic assessment of low-grade neuroepithelial lesions.

**Supplementary Information:**

The online version contains supplementary material available at 10.1186/s40478-025-02209-3.

## Introduction

Polymorphous low-grade neuroepithelial of the young (PLNTY) tumors have recently been described as rare epileptogenic tumors affecting the pediatric population and young adults [1, 2]. PLNTYs often exhibit dominant oligodendroglioma-like structures, but also contain more infiltrative astrocytic-like structures, as well as areas with high calcification. These tumors are carrying activating genetic alterations of either B-Raf proto-oncogene (*BRAF* V600E, referred now on as BRAF_V600E_ ) or fibroblast growth factor receptor 2 or 3 (*FGFR2/FGFR3*) and therefore are known to activate the mitogen-activated protein kinase (MAPK) pathway [1, 3–5]. PLNTYs do not carry isocitrate dehydrogenase (*IDH1/2*) mutations and are 1p/19q co-deletion negative, distinguishing them from adult-type diffuse gliomas [2]. Both PLNTYs and oligodendrogliomas are known to exhibit oligodendrocyte transcription factor 2 (OLIG2) and glial fibrillary acidic protein (GFAP) positivity, but as a distinguishing feature, PLNTYs do not exhibit neuronal differentiation. Expression of neuronal markers, such as neuronal nuclear protein (NeuN), remains negative in PLNTY tumors [6, 7]. Another distinctive and thus diagnostic feature of PLNTYs is positive stain of CD34, which is a transmembrane phosphoglycoprotein expressed during the development of the nervous system in progenitor cells, but not typically present in the mature brain nor in oligodendroglioma and astrocytic-like tumors [6–9]. Methylation profiling separates PLNTYs from other pediatric low-grade neuroepithelial tumors with the highest resemblance to gangliogliomas [1]. Reported PLNTY cases are non-aggressive tumors [1–3], with the exception of a single case reported carrying a *FGFR3::TACC3* fusion and undergoing a malignant transformation to a higher-grade glioma [10]. Epithelioid GB (E-GB) is a rare morphological variant of the most common primary malignant brain tumor GB. Representing a recently defined entity, E-GBs are often discovered in young adults and associated with extremely poor prognosis of only a median of ten months survival after the diagnosis [2, 11–14]. It is histologically characterized by its epithelioid, rhabdoid, or even melanoma-like cells, including sizable vesicular and prominent nuclei as well as abundant cytoplasm [2, 14]. Wide necrotic areas are also common in E-GB pathology [14–16]. E-GB tumors are typically negative for *IDH* mutations, but approximately 50% are positive for BRAF_V600E_[14–16] which has an activity-enhancing effect on the MAPK pathway, increasing proliferation and migration of the tumor cells. Furthermore, telomerase reverse transcriptase (*TERT*) promoter mutation, O-6-methylguanine-DNA methyltransferase (*MGMT*) promoter methylation, homozygous deletion of cyclin dependent kinase inhibitor (*CDKN2A/B*) as well as overexpression of enhancer of zeste 2 polycomb repressive complex 2 (*EZH2*) are common in E-GB [13–17]. BRAF_V600E_is also commonly found in lower-grade tumor types such as pleomorphic xanthoastrocytomas (PXAs) (66%), PXAs with anaplastic features (65%), gangliogliomas (18%), anaplastic gangliogliomas (50%) and extra cerebellar pilocytic astrocytomas (9%) [12]. E-GBs have been reported to resemble PXAs by genetic and histologic features as well as by their methylation profile [18, 19], but because it is only a recently characterized entity, more genome-wide profiling is still warranted. Typically, E-GB arises as a primary tumor, but in rare cases, it has been reported also to arise from low-grade PXA [13, 20] and to co-localize with other low-grade gliomas [13, 21, 22]. In most of the reported cases, E-GB is carrying the BRAF_V600E_.

In this study, we describe clinical and genetic data from a single patient presenting a novel finding of PLNTY-like tumor and epithelioid GB tumor simultaneously. We aimed to uncover any chromosomal changes and the somatic, oncogenic mutations that have been driving the evolution of these tumors. Furthermore, we aimed to expose the possible predisposing germline variants that could explain the formation and co-existence of two rare tumor types in the index patient, with a familial history of brain tumors.

## Case presentation: Materials and methods

### Tumor samples

The tumor samples were obtained from the index patient (44 years old) during surgery and autopsy at Tampere University Hospital Finland. Freshly frozen (FF) tumor tissue embedded in Tissuetek O.C.T (Sakura Finetech, Japan), formalin-fixed paraffin-embedded (FFPE) tumor tissue, and patient-derived cell line (CL) for culturing were obtained from the epithelioid glioblastoma (E-GB) tissue shortly after surgery. From a PLNTY-like tumor FFPE tissue was obtained in the autopsy. The PLNTY-like tumor was highly calcified. For the removal of calcium deposits, decalcification was performed using a 5% formic acid solution at room temperature. Decalcification was performed under continuous monitoring of the hardness of the specimen. In addition, blood samples were collected from the patient and her mother in EDTA tubes. Comprehensive differential diagnosis was established based on histological and molecular findings for both tumors by several experienced neurosurgeons and neuropathologists at Tampere University Hospital, Finland.

**Fig. 1:**
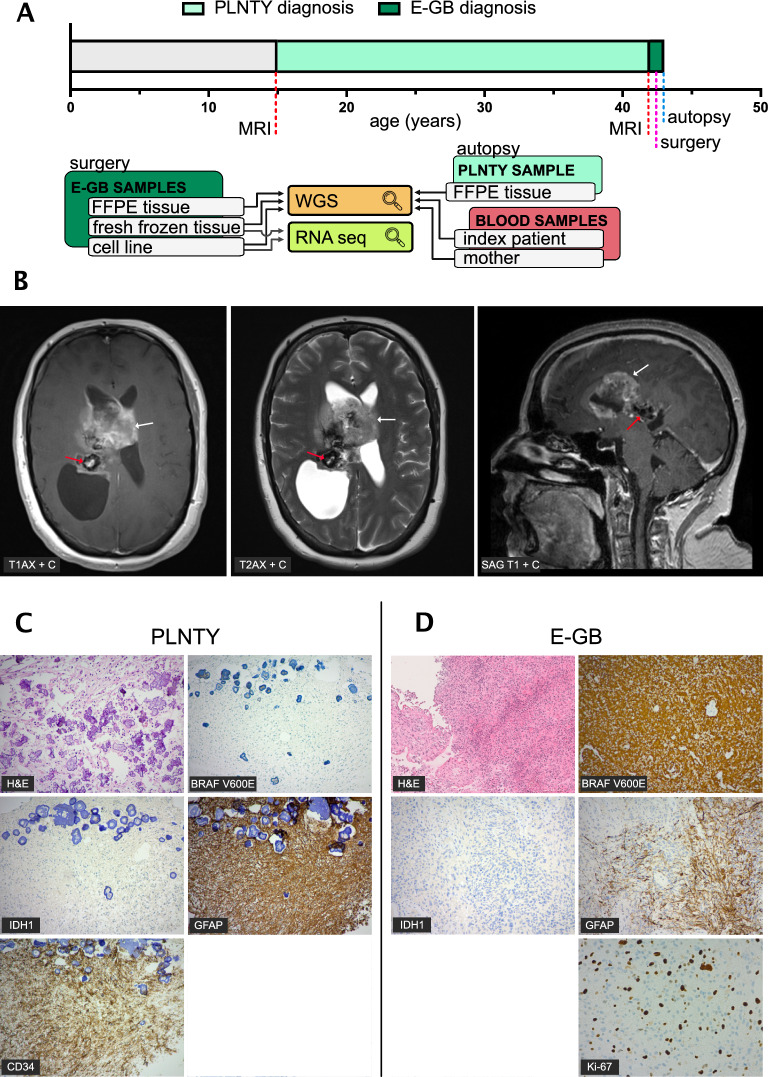
Two distinctive tumors in the same patient showed clear differences in the stain patterns of H&E and common immunohistochemical markers. **A** Timeline of detection and progression of PLNTY-like tumor to E-GB, as well as presentation of samples collected from each tumor. All the tumor samples (FFPE sample from the PLNTY and FFPE, fresh frozen (FF) tissue and cultured cell line (CL) samples from the E-GB) were subjected to WGS. In addition, E-GB FF tumor tissue and cell line samples were analyzed with RNA sequencing. **B** Magnetic resonance imaging (T1 weighted axial with gadolinium, T2 weighted axial with gadolinium, and T1 weighted sagittal with gadolinium) revealed two distinctive tumor masses: a larger tumorous mass (E-GB, red arrow) located in spatial proximity of the smaller tumorous mass (PLNTY-like tumor, white arrow). **C** Immunohistochemical stain of the PLNTY-like tumor revealed prominent confluent calcification and infiltrative growth pattern combined with oligodenroglial or astrocytic mildly atypical cells (H&E). BRAF_V600E_ and IDH1 p.R132H mutant protein stains were negative, but the tumor showed strong CD34 positivity along with asymmetrical GFAP positivity. Ki-67 proliferation index was low. **D** Immunohistochemical stains of E-GB tumor shows a highly cellular tumor consisting of epithelioid atypical glial cells (H&E). BRAF_V600E_ stain was highly positive. The tumor cells were negative for IDH1 p.R132H and for CD34. GFAP immunostain showed patchy positivity. Ki-67 immunostain shows a high labeling index for proliferation (15–20%)

### Immunohistochemical analysis

All immunohistochemical stains were performed in Fimlab laboratories, at Tampere University Hospital Finland. Hematoxylin and eosin (HE) stains were executed with Sakura Tissue-TEK Prisma using a progressive staining method. Sakura RTU staining reagents (#9130-E) were used for the HE stain. All the other stains (BRAF_V600E_, IDH1 R132H, GFAP, CD34, Ki-67) were performed with Ventana Benchmark Ultra (Roche, Tuscon) using immunoperoxidase staining method (Supplementary Table S1). Antigen retrieval was performed with Ventana Benchmark Ultra CC1 solution with specific incubation times and temperatures for all proteins (Supplementary Table S1).

### Cell line preparation

Tissue from the E-GB tumor was processed for the cultivation of a cell line right after the operation. Visibly necrotic tissue areas were initially removed from the sampled tissue and the remaining tissue was diced with a scalpel. Next, the tumor mass was lysed with enzymatic digestion using a tumor dissociation kit (#130-095-929, Miltenyi Biotech). Finally, the single cell suspension in DMEM/F12 (#21,331, Gibco) was filtered through a 70 µm cell strainer. The suspension was pelleted and resuspended in NSC (neural stem cell) media (DMEM/F12, DMEM Neurobasal medium (#21,103,049, Gibco), B-27 supplement (#17,504,044, Gibco) and N-2 supplement (#17,502,048, Gibco)). The cell suspension was diluted in two different growth media: NSC media including FGF (#100-18B, Peprotech) with EGF (#AF-100–15, Peprotech) and MEM-FBS media (MEM (#M2279 Sigma), 10% Fetal bovine serum (#10,270,106, Gibco) and GlutaMax (#35,050,061, Gibco). The NSC media culture was discarded one month after the initiation, because it did not exhibit signs of cell growth. The MEM-FBS cell line started to form spheres after two weeks of culturing and the first vial of the cells was frozen after one month of culturing.

### Whole genome sequencing (WGS)

DNA was isolated from the blood samples of the index patient and mother using QIAamp DNA Blood Mini Kit (#51,104, Qiagen). DNA from tumor tissues was obtained from FFPE (PLNTY, E-GB) using GeneRead DNA FFPE Kit (#56,404, Qiagen) and FF tissue (E-GB). Cell line DNA sample was prepared using p8 (E-GB) with QIAmp DNA Mini Kit (#51,304, Qiagen). DNA extracted from PLNTY-like tumor and E-GB FFPE samples were further purified with 1,3 × AMpure XP beads (#A63881, Beckman Coulter).

Library preparation and WGS were performed by Novogene with Illumina HiSeq technology paired-end 150 bp sequencing (PE150), with 30X coverage for the blood samples and 60 × coverage for the FFPE, FF tissue, and CL.

### RNA sequencing

RNA was extracted from the E-GB FF and CL (p9) samples with mirVana isolation kit (#AM1560, ThermoFisher Scientific). Library preparation and RNA sequencing were performed by Novogene with Illumina HiSeq technology paired-end 150 bp sequencing (PE150) 16 Gb raw data per sample. RNA sequences were aligned to reference genome assembly GRCh38 using star (version 2.7.10a).

### DNA methylation

Genome-wide DNA methylation profiling of DNA isolated from eGB CL and FFPE tumor samples was performed using an Illumina Infinium HumanMethylation EPIC Kit. DNA methylation-based molecular classification was performed similarly as previously described [23] using two versions (older and latest version) of an in-house Heidelberg Brain Tumor Methylation Classifier. The latest version (version 12.8) is trained using 7,495 methylation profiles, including 184 methylation subclasses representing different central nervous system tumor and control entities [24]. The analyzed samples were visualized together with relevant reference samples using t-SNE (t-distributed stochastic neighbor embedding) and UMAP (Uniform Manifold Approximation and Projection). Unfortunately, we did not have enough DNA or material left from the PLNTY FFPE sample for DNA methylation analysis.

### Karyotyping

E-GB cell line (p9) karyotyping was performed as a service in Fimlab (Tampere University Hospital, TAYS). For the analysis cells were cultured on a laminin coated primaria plate in MEM-FBS media for 7 days during which it was passaged once. Karyotyping analysis was used as a reference for the copy-number profile analysis (Supplementary Table S2).

### Variant calling and somatic copy-number profile analysis

WGS data was aligned to reference genome assembly hg38 using bwa-mem [25]. Aligned paired-end read variant calling was performed using GATK 4.1.8.1, following GATK best practices guidelines. Somatic variants were analyzed using GATK/Mutect2 [26] and germline variant analysis was performed using GATK/Haplotypecaller [26]. Somatic SNVs and indels were required to have at least 3 supporting reads and 10 total reads based on alleleCount v.4.0.2 (https://github.com/cancerit/alleleCount) and Mutect2, respectively. In addition, SNVs were filtered based on FFPEsig [27] to remove the effect caused by formalin fixation. Genome copy number segmentation analysis of somatic copy number alterations (CNAs) was performed using Battenberg [28] and the detected copy number segments were further refined to find the best-suited copy number profile using CN-solver (manuscript under preparation). Furthermore, karyotyping of E-GB cell line was used as a reference for determining the correct copy numbers (Supplementary Table S2). Structural variations (SVs), including possible gene fusions, were analyzed with Svaba [29]. SVs detected only in all three E-GB samples and SVs shared by all E-GB samples and PLNTY-like tumor were analyzed further. Each detected copy number segment in the genome-wide loss of heterozygosity (LOH), that was discovered in both studied tumors, was analyzed to determine the ratio of single nucleotide polymorphisms (SNPs) inherited from either of the parents using in-house software made for the specific purpose of conducting the analysis.

For further analysis, we selected somatic variants that were detected in PLNTY-like tumor and all three E-GB samples (FF, FFPE, and CL), or only in all three E-GB samples. Point mutations were prioritized based on the Cosmic database [30] annotations, ClinVar [31] pathogenicity annotations, and functional annotations. For the genes reported in the manuscript, the main transcript carrying the variant was selected based on NCBI first isoform.

### Germline analysis

For the search and analysis of brain tumor susceptibility mutations, a population frequency-based filtering was employed using gnomad 3.1 data [32], excluding variants found at ⫺10% frequencies in any population in the database. Only variants shared between mother and the index patient were selected for the analysis. Variants detected in the chromosomes solely inherited from the father in the tumor samples were excluded from the analysis (chr 1–3, chr6, chr9, chr 11–12, chr 14–16 and chr22). Variants for further analysis were selected if they were detected either in ClinVar [31] pathogenicity annotation or Cosmic database [30] (variant reported > 1 in the database). All remaining variants were filtered based on algorithmically predicted pathogenicity (SIFT, SIFT 4G, LRT, MutationTaster, MutationAssessor, FATHMM, PROVEAN, MetaSVM, MetaLR, MetaRNN, and M-CAP), so that variants were predicted to be deleterious > 3 times. Lastly, the remaining variants were prioritized based on functional annotations. For the genes reported in the manuscript, the main transcript carrying the variant was selected based on NCBI first isoform.

## Results

### Clinical presentation and pathological evaluation revealed two district tumor types sharing similarities in GFAP stain

During adolescence at the age of 17, a benign calcified tumor mass had been detected in a female patient suffering from headache with magnetic resonance imaging (MRI) of the central nervous system (CNS). Imaging revealed a lesion that was calcified and did not have contrast enhancement. The radiological diagnosis was uncertain at the time, but plexus papilloma was proposed. The patient was first followed by serial imagining during a four-year follow-up time and since the tumor showed no signs for growth or evolution, the imagining was discontinued. Because the tumor was considered benign and stable, no surgery or treatment was performed. Approximately 27 years later, the patient (at the age of 44) started to suffer from nausea, vomiting, headaches, and weakness of upper and lower limbs on the left side. A new MRI of the CNS revealed a large tumor mass located near the smaller, previously detected, benign, and calcified tumor mass (Fig. 1A & B).

Subsequently, craniotomy, tumor resection, and tumor tissue sampling were performed and based on a histopathological analysis, diagnosis of E-GB (grade IV) was established. The tumor tissue was widely necrotic and highly cellular, with atypical epithelioid cells and pleomorphic nuclei accompanied with multiple mitotic structures (Fig. 1C). The tumor was negative for *ATRX* and *IDH1*/*IDH2* mutations. Strong positive stains for BRAF_V600E_ and S100 were detected, as well as patchy stain of GFAP, which are all common characteristics of E-GB (Fig. 1C). Stain of p53 was WT-like (15–20%) and proliferative activity was 15–20% when estimated with Ki-67 stain (Fig. 1C). Molecular analysis revealed the tumor carrying BRAF_V600E_ mutation (Supplementary Table S3).

Postoperatively, the patient developed hemiparesis, and the level of consciousness remained low. The follow-up was performed first at the intensive care unit and then at the neurosurgery ward, where the patient died a week later after the craniotomy. The epithelioid glioblastoma had resulted in a large intracerebral hemorrhage and significant cerebral edema, although no definitive signs of herniation were observed. An autopsy was performed leading to a discovery of two clearly separate tumor masses within close spatial proximity of each other. The hemorrhage was considered a plausible cause of death. Additionally, a recent 4 cm cerebral infarction was identified in the right frontal lobe.

The more recent lesion, and the larger of the two tumors (spanning 7 × 6.5 cm in diameter), was located in the both lateral ventricles, predominately on the right side. The tumor was also seen in the corpus callosum area, and it infiltrated subcortically on the right (Fig. 1B). The tumor mass was highly cellular with epithelioid atypical cells, corresponding to the diagnosis of E-GB (grade IV) which is a descriptive GB subtype and does not form a diagnostic class of its own [2, 33]. CD34 stain was negative for the E-GB tumor, in concordance with previous reports [10]. DNA methylation analysis of E-GB (FFPE and CL) was performed twice: using an older model and by rerunning the analysis with a most recent model (v12.8). In the first analysis, both E-GB FFPE and CL samples were classified as IDH wildtype GBs (methylation class GBM, RTK II) with a moderate confidence (score 0.65 and 0.48, respectively). When using the updated model (v12.8.), both samples were classified into subclass (anaplastic) pleomorphic xanthoastrocytoma (PXA) (confidence score 0.98 and 0.63, respectively), positioning the E-GB tumor into a prognostically more favourable PXA-like E-GB subclass [19]. PXA-like E-GBs are predominantly diagnosed in children and young adults, typically carry BRAF_V600E_ mutation, and epigenetically resemble PXAs based on their DNA methylation profile [19, 33]. Interestingly, the analyzed E-GB samples were positioned next to GBM RTK II and PXA samples in the t-SNE and UMAP visualizations, respectively (Supplementary Fig. S1).

The other, earlier tumor, was smaller in comparison (4.5 cm in diameter), with widely calcified and yellowish tumor mass with glial, mostly oligodendroglial-like cells. It was located in the deep parietal region with extension into the lateral ventricle, which is atypical for classical oligodendroglioma but consistent with certain low-grade glial or glioneuronal neoplasms. Immunohistological analysis revealed strong CD34 positivity, along with strong and patchy GFAP positivity (Fig. 1C). IDH1 R132H, synaptophysin, and BRAF_V600E_ stains were negative (Fig. 1C). However, molecular analysis revealed BRAF_V600E_ mutation (Supplementary Table S3). DNA methylation analysis was not performed, as we did not have enough isolated DNA or tumor material left for the analysis. Furthermore, the PLNTY-like tumor (FFPE) had been decalcified, which further compromises the DNA quality and hinders analysis with a low sample input. However, comprehensive differential diagnosis was performed. Relevant differential diagnoses for this tumor type include oligodendroglioma, which was not supported due to the IDH-negative status, CD34 positivity, and the presence of a BRAF_V600E_ mutation, all of which are atypical for oligodendroglioma. Pilocytic astrocytoma is unlikely given the absence of classic features, such as Rosenthal fibers or eosinophilic granular bodies, and BRAF fusions are more typical than point mutations in that entity. Subependymoma may be considered based on location but is generally hypocellular and CD34-negative. Papillary glioneuronal tumour and rosette-forming glioneuronal tumour are both excluded based on synaptophysin negativity, which is uncharacteristic for these glioneuronal neoplasms. Central neurocytoma could be considered based on location, but the immunophenotype is inconsistent. Central neurocytomas are typically synaptophysin-positive and GFAP-negative, and they do not harbor *BRAF*_*V600E*_ mutations. While PLNTY and MAPK pathway altered diffuse low-grade gliomas, like PXA may share MAPK-related genetic alterations such as BRAF_V600E_ , PLNTY can be distinguished by its prominent calcifications, CD34 expression, and characteristic morphology. Histologically the tumor lacked pleomorphism, multinucleated giant cells, xanthomatous change, eosinophilic granular bodies, necrosis, and mitotic activity, which are all typical for PXA. Furthermore, *CDKN2A/B* loss or inactivation through other genetic alterations was not identified in the tumour, which strongly argues against PXA. Based on World Health Organization’s (WHO) 5th edition, differential diagnosis resting on the histological appearance, immunoprofile, molecular findings, and anatomical location of the tumor, the diagnosis is most consistent with PLNTY [2]. Because the methylation analysis was not done to this tumor due to insufficient DNA amount and quality, we will refer to the tumor as PLNTY-like tumor. Based on the neuropathological examination, the two tumors were nearly contiguous. The intervening tissue between the tumors was not specifically sampled.

### Both tumors carried a nearly genome-wide loss of heterozygosity

**Fig. 2:**
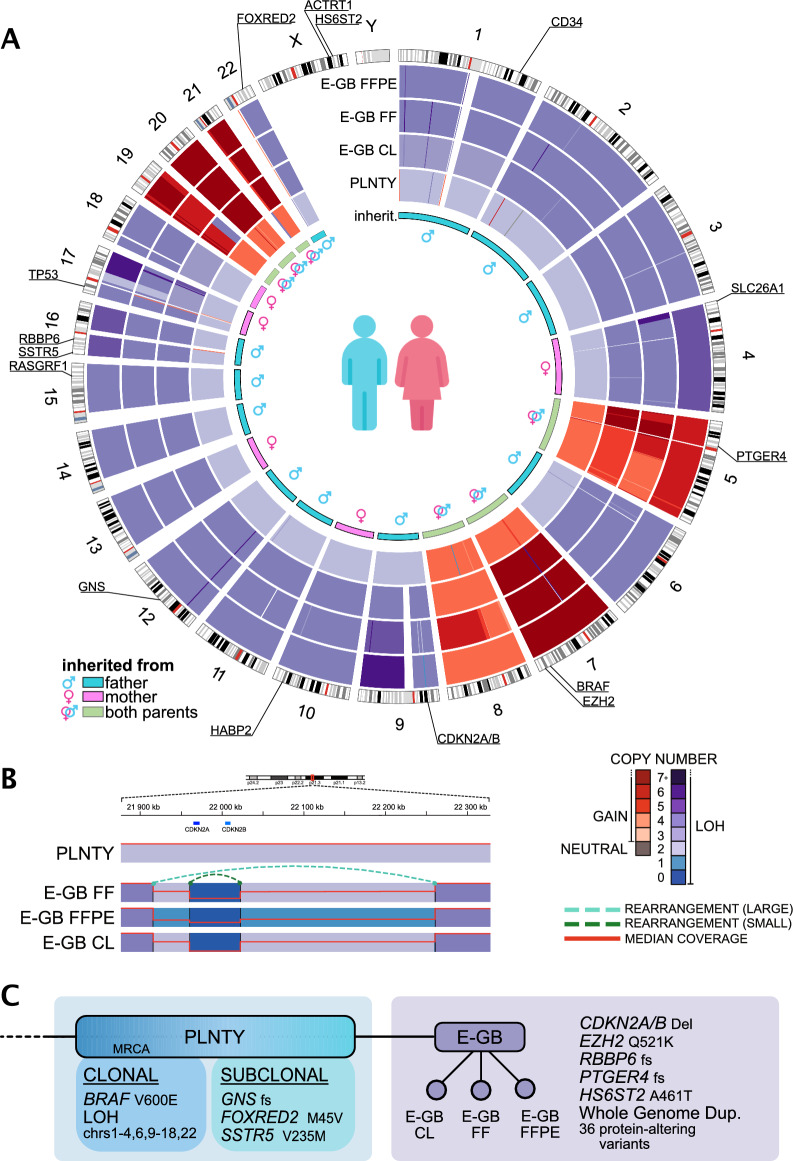
Both tumors carried a prominent, nearly genome-wide loss of heterozygosity, indicating a common origin. **A** Circos plot presenting the copy number changes throughout the chromosomes in the PLNTY-like tumor (representing formalin fixed paraffin embedded (FFPE) tissue sample) and three E-GB samples: E-GB FFPE tissue, fresh frozen tissue (FF), and patient-derived cell line (CL). Copy neutral loss of heterozygosity is observed in chromosomes 1–4, 6, 9–18, and 22. In the E-GB samples, genome-wide WGD has duplicated CN-LOH regions and also resulted in highly amplified copy number state of chromosomes 5, 7–8 and 19–21. **B** Two overlapping deletions of *CDKN2A/B* gene locus were detected in all E-GB samples. Breakpoints and rearrangements of two deletions are marked in the figure (larger deletion with light green and smaller deletion with dark green dash line). The most prevalent CN was 2 + 0 in the locus of the larger deletion in two E-GB samples (FF and CL) and 1 + 0 in FFPE E-GB sample. Smaller deletion was homozygous in all the E-GB samples. *CDKN2A/B* locus in the PLNTY-like tumor sample was still intact, suggesting the deletion of *CDKN2A/B* driving the malignant transformations of the tumor. The red line indicates the relative median read coverage in the locus including also the reads originating from normal cells in tissue samples. **C** A cladogram showing the relationship and shared genetic, oncogenic features between the two tumors, indicating a monoclonal origin. Based on the identified oncogenic drivers (most prominently, LOH and BRAF_V600E_ ) that are shared between the two tumor types, an ancestral form (MRCA, most recent common ancestor) of the E-GB tumor can be established to be the PLNTY-like tumor

WGS-based copy number analysis of the PLNTY-like tumor (FFPE) and three E-GB samples (FF, FFPE, and CL) revealed an unusual copy-number profile in all tumor samples. In the PLNTY-like tumor, 16/22 autosomal chromosomes were discovered to have lost one parental allele, resulting in a copy-neutral loss of heterozygosity (CN-LOH) for the entire chromosome. The notable exceptions were chr5, chrs 7–8, and chrs 19–21 that were present in four copies (Fig. 2A, Supplementary Fig. S2). This copy number profile is consistent with an early catastrophic event already during the formation of the PLNTY-like tumor, where 16 chromosomes have lost an allele (LOH) that has been replaced by an identical copy during a first whole genome duplication (WGD) event settling them into a CN-LOH state. The same WGD event has affected the remaining six chromosomes with retained heterozygosity (chrs 5,7,8 and 19–21), doubling their copy number to four. The mutation allele fractions in the CN-LOH-affected chromosomes are also corroborating the very early nature of the LOH and WGD events in the formation of the PLNTY-like tumor. Mutations in both of the identical copies of the CN-LOH chromosomes were discovered to be virtually non-existent, indicating that very few mutations had occurred before the first WGD event. Later during the evolution of PLNTY-like tumor into E-GB, another WGD event has occurred doubling those 16 CN-LOH affected chromosomes, resulting in a predominantly tetraploid genome. Six chromosomes (chr5, chrs 7–8 and chrs 19–21) that in the early events of the PLNTY-like tumor development had remained in normal diploid copy number, gained additional copies during the tumor evolution into E-GB, settling them into a highly amplified state (Fig. 2A, Supplementary Fig. S2). In previous studies, copy number gains in chr7 and chr19 have been commonly observed in GB [34]. Furthermore, WGD is a recurrently observed, early event in GB [35, 36].

The prominent LOH in each sample allowed us to investigate whether there was a preference or a pattern to the familial ancestry of the lost alleles. By analyzing the SNPs in each LOH-affected continuous copy number segment, we were able to determine if the remaining copy was of maternal or paternal ancestry. We discovered each of the studied segments to exhibit > 95% matching SNPs with one of the parents, and all segments within a chromosome to match with the same parent. This finding suggests that the loss of one parental copy in the LOH-affected chromosomes has occurred while the chromatid is intact, in contrast to a catastrophic or commonly recurring breaking and consequent mis-repairing of the genomic material. In total, 11 of the 16 LOH-affected chromosomes (chrs1-3, chr6, chr9, chrs11-12, chrs14-16 and chr22) were found to have paternal inheritance and the remaining 5 chromosomes (chr4, chr10, chr13, chrs17-18), maternal inheritance (Fig. 2A). The 6 autosomal chromosomes that were not affected by LOH (chr5, chr7, chr8 and chr19-21) had only small segments (< 5 Mb) with one-copy losses that are commonly seen in various different types of cancer.

### PLNTY-like tumor and E-GB tumors share a common ancestor

In order to identify oncogenic drivers underlying PLNTY-like tumor evolution and its eventual progression to E-GB, we performed a DNA analysis of the somatic single nucleotide variants (SNVs) and short insertions and deletions (indels) on the WGS samples (PLNTY-like tumor (FFPE) and three E-GB (FFPE, FF, and CL)). As both decalcification and formalin treatment have been shown to cause DNA damage [27, 37, 38], we based the analysis on variants detected in the E-GB FF sample (Fig. 1A) [27]. Altogether, 233 mutations including a known oncogenic driver mutation in *BRAF* (substitution of T > A leading to amino acid substitution of valine to glutamic acid (chr7, exon15: c.T1799A:p.V600E in transcript NM_001354609)) [1, 3, 14–16] were detected in E-GB FF and found to be shared between PLNTY-like tumor and all other E-GB samples, indicating a monoclonal origin and common ancestry for the two tumors. In addition to oncogenic BRAF_V600E_, three other mutations affecting protein coding-regions were shared between PLNTY-like tumor and E-GB: non-synonymous SNVs in FAD dependent oxidoreductase domain containing 2 ***(FOXRED2)*** and in somatostatin receptor 5 (*SSTR5*), and a frameshift deletion in glucosamine (N-acetyl)-6-sulfatase (*GNS*) (Supplementary Figs. S3, S4, Supplementary Table S3). Non-synonymous *FOXRED2* carried an A > G substitution leading to a methionine to valine substitution (chr22, exon2: c.A133G:p.M45V in transcript NM_001102371) and *SSTR5* carried G > A substitution leading to a change of valine to methionine (chr16, exon1: c.G703A:p.V235M in transcript NM_001053). Both *FOXRED2* and *SSTR5* variants were subclonal in the PLNTY-like tumor: they were present in 12% and 7.0%, respectively, of DNA reads, meaning their presence in cancer cells was approximately 70% and 40% considering the sample purity (33.5%) and copy numbers. RNA expression of these mutations was examined in E-GB FF tissue and E-GB CL sample. BRAF_V600E_ and *FOXRED2* SNVs were detected in 21/54 and 46/79 RNA-seq reads in the FF tissue and 32/69 and 153/306 reads in the cell line sample, respectively (Supplementary Table S3), thus both genes showing mutation in half of the reads, as expected. *SSTR5* was not expressed at the RNA level in E-GB. The *BRAF* locus is on chromosome 7, within a high chromosomal gain region detected in the CNV analysis of the E-GB samples. This finding suggests an amplification of the effect of the oncogenic BRAF_V600E_ [39]. Consistently, strong BRAF protein expression was corroborated by the immunohistochemical BRAF_V600E_ stain of the E-GB sample (Fig. 1D).

Frameshift deletion of *GNS* caused a change of aspartic acid to cysteine (chr12, exon5: c.616_617del:p.D206Cfs*3 in transcript NM_002076) in exon 5/14 and led to a premature stop codon three amino acids later at the beginning of exon6. *GNS* frameshift deletion was subclonal in the PLNTY sample (DNA allele frequency 6.5%, corresponding fraction in cancer cells approximately 40%) and more prevalent in E-GB samples (DNA allele frequency 52%, 44%, and 39% for FFPE, FF, and CL, respectively, corresponding to 100% presence in cancer cells considering sample purities 73.5%, 77%, and 100% and copy numbers). RNA analysis revealed that the *GNS* frameshift was expressed by only 4/385 reads in the FF tissue and 8/1419 in the CL sample (Supplementary Table S3), suggesting a destabilizing effect on the RNA or an allele expression imbalance. *FOXRED2, SSTR5* and *GNS* are located in chromosomes 22, 16 and 12, respectively, in regions affected by LOH in both PLNTY and E-GB samples. To conclude, all the nonsynonymous point mutations shared by the PLNTY and all three E-GB samples, except BRAF_V600E_, were subclonal in PLNTY-like tumor and clonal in E-GB, suggesting that they have been present in the ancestor clone of E-GB. Of these, only BRAF_V600E_ is known to be oncogenic, and only BRAF_V600E_ and *FOXRED2* variants were properly expressed in E-GB samples.

### The loss of *CDKN2A* tumor suppressor driving the E-GB progression

To better understand the progression of PLNTY-like tumor into E-GB, we analyzed structural variants (SV) in our samples. Overall, we detected 13 SVs shared between three E-GB tumor samples (FFPE, FF and CL) and the PLNTY sample (Supplementary Table S4). In total 30 SVs were shared between three E-GB samples but were not detected in the PLNTY-like tumor (Supplementary Table S4). Interestingly, we detected two separate focal deletions overlapping in the *CDKN2A/B* gene locus in the E-GB samples (Supplementary Table S4). *CDKN2A* codes for the tumor suppressor gene p16, which is a critical controller of G1/S cell cycle checkpoint and associated with increased cell proliferation [40]. CN analysis suggests that both deletions have occurred prior to the second WGD in E-GB samples yielding to CN 2 + 0 around the locus of larger deletion and homozygous deletion in the locus of the smaller deletion (Fig. 2B). *CDKN2A* and *CDKN2B* genes did not carry any somatic point mutations in any of the samples. Neither of the focal *CDKN2A/B* deletions was detected in the PLNTY-like tumor, suggesting that the *CDKN2A/B* deletions have contributed to the progression of PLNTY-like tumor into the E-GB. Furthermore, there was only one copy of the wider *CDKN2A/B* deletion in the E-GB FFPE sample, which could arise *e.g.* via homologous recombination-mediated processes similarly as in copy-neutral LOH events [41].

### *EZH2* Q521K and 44 other exonic point mutations specific to E-GB

We next investigated somatic point mutations that were detected only in the E-GB samples to reveal possible aberrations that have participated in the malignant transformation of the tumor. In total, 3808 somatic point mutations were shared between all three E-GB samples but not detected in the PLNTY-like tumor. From these mutations, 45 were affecting exonic regions (4 frameshift deletions, 28 nonsynonymous, and 13 on non-coding RNAs) (Supplementary Figs. 3, 4, Supplementary Table S3). Two of these SNVs stood out in Cosmic database [30] annotations, ClinVar [31] pathogenicity annotations, functional annotations, and in RNA expression analysis. Firstly, *EZH2*, which is commonly overexpressed in E-GB [15, 17], carried a nonsynonymous SNV of C > A substitution leading to glutamine_521_ substitution to lysine (chr7, exon14:c.C1561A:p.Q521K in transcript NM_001203247) (Fig. 2C). *EZH2* is located in chromosome 7, with high chromosomal gains in the E-GB samples, supporting the possible overexpression of *EZH2* in our samples. Q521K is previously unreported but it is predicted to be pathogenic in the AlphaMissense database [42] and by 7/10 prediction algorithms (Supplementary Fig. 5). The *EZH2*_Q521K_ point mutation presents as clonal in E-GB when considering the sample purities, high DNA copy numbers in this genomic region, and the fraction of reads carrying it: 18–32% in DNA samples and 16–17% in RNA samples (Fig. 2C, Supplementary Table S3). Secondly, heparan sulfate 6-O-sulfotransferase 2 (*HS6ST2*), which catalyzes the transfer of sulfate to heparan sulfate, participating in multiple cellular functions, carried a nonsynonymous SNV (chrX, *e*xon6:c.G1381A:p.A461T, in transcript NM_001077188) in 31–39% of DNA reads, and was expressed in all RNA reads detected in FF tissue (47/47) and CL (15/15) (Fig. 2C, Supplementary Table S3). Furthermore, four frameshift deletions were detected in all E-GB samples; namely in genes ras protein specific guanine nucleotide releasing factor 1 (*RASGRF1),* RB binding protein 6, ubiquitin ligase *(RBBP6),* prostaglandin E receptor 4 *(PTGER4),* and actin related protein T1 *(ACTRT1)*, from which only *RBBP6* (chr16, exon18:c.5286_5287del:p.H1763Q in transcript NM_006910) and *PTGER4* (chr5, exon3:c.1344_1345del:p.S450C in transcript NM_000958) mutations were expressed in the RNA data (50–85% and 25–81% of the RNA reads carried the frameshift deletion, respectively). *RASGRF1 (*with mutation chr15, exon16:c.2497_2498del:p.S833 in transcript NM_001145648) and *ACTRT1* (with mutation chrX, exon3: c.1344_1345del:p.S450C in transcript NM_138289) genes were not expressed at all in the E-GB RNA samples (Supplementary Table S3).

### Predicted predisposing genetic risk variants

Because the patient had a family history of brain tumors on the mother’s side, we performed WGS from blood-derived DNA samples and compared possible predisposing germline variants shared between the index patient and her mother. The copy number analysis revealed prominent LOH throughout the index patient’s tumor genomes. An analysis of inheritance of SNPs present in LOH-affected chromosomes (chrs1-4, chr6, chr9-18 and chr22) revealed that the chromosomes were inherited solely from one parent only (Fig. 2A). For further analysis of the predisposing risk variants, we selected variants that were present in the tumor and inherited from the mother, including also genomic regions with preserved heterozygosity in the PLNTY-like tumor (altogether chrs 4–5, chrs 7–8, chr10, chr13, and chrs 17–21) (Fig. 2A). Analysis of Cosmic database [30], ClinVar [31] pathogenicity and algorithmically predicted pathogenicity did not reveal any known high-risk variants, however two variants inherited from the mother were found to be more interesting based on their functionality. Hyaluronan binding protein 2 (*HABP2*), which is an extracellular serine protease that binds hyaluronic acid, carried nonsynonymous SNV leading to substitution of glycine to glutamic acid (chr10, exon13:c.G1601A:p.G534E in transcript NM_004132) (Supplementary Table S5). *HABP2* has been suggested to work as a tumor suppressor, and mutations in the gene can lead to uncontrolled cell proliferation and migration [43]. However, the *HABP2* gene was not expressed in RNA extracted from the E-GB tumor. The second variant of interest, solute carrier family 26 member 1 (*SLC26A1*), which is an anion exchanger expressed in multiple tissues including brain, carried a nonsynonymous SNV causing a change of alanine to threonine (chr4, exon2:c.G166A:p.A56T in transcript NM_022042). The *SLC26A1* variant is reported to carry unknown significance in the ClinVar database [31] and predicted to be damaging by nine prediction algorithms. Furthermore, it was expressed in all RNA reads detected in FF tissue (61/61) and CL (42/42) (Supplementary Table S5).

## Discussion and conclusions

This study elucidates the genetic mechanisms related to progression of PLNTY tumors, that are typically non-aggressive, indolent tumors. We observed the wide loss of heterozygosity followed by WGD in the histologically and molecularly supported PLNTY, accompanied by focal deletion of *CDKN2A/B* and additional WGD in the E-GB. A similar rare genome-wide LOH has been previously detected by Nordfors et al*.* [44] in a case study of a child with atypical teratoid/rhabdoid tumor (AT/RT) and mother with an astrocytoma tumor.

The DNA methylation profiling of E-GB tumor samples position the E-GB tumor into the PXA-like E-GB subgroup when using the most recent Heidelberg DNA methylation classifier. Although we had earlier classified the analyzed E-GB samples into the GBM RTK II class, representing IDHwt GBM‐like E-GB group, higher confidence was received with the latest classifier, and the tumor subtype was defined accordingly. Also in the t-SNE and UMAP visualizations, the analyzed E-GB samples localized next to GBM RTK II and PXA tumors, respectively. These discrepancies might relate to the atypical origin or genomic landscape of our E-GB tumor, and it is possible that the tumor harbours DNA methylation features typical for both GBM RTK II and PXA tumors. Different classification models and visualization algorithms might weight the included features differently, which leads to differences in their output, including the confidence score of the classification.

PXA-like E-GBs are associated with better survival than IDHwt GBM‐like or RTK1 pediatric GBM‐like neoplasms [19]. However, several histological features in the analyzed E-GB tumor were indicative of tumor aggressiveness, including wide necrosis, high cellularity, pleomorphic nuclei, and presence of multiple mitotic structures. Ki-67 stain-based proliferative activity was 15–20%. Due to insufficient DNA amount and quality, the diagnosis of PLNTY-like tumor was not confirmed with methylation studies, which is a clear limitation of this study. However, in the comprehensive differential diagnosis performed by several experienced neurosurgeons and neuropathologists, the histopathological, immunohistochemical, molecular, and radiological features of the original tumour was the most consistent with PLNTY diagnostic. Furthermore, no *CDKN2A* losses were identified in this tumour, which also supports the PLNTY diagnosis.

In our study, chr7 had acquired additional copies (representing 8 + 0 copies) in a PXA-like E-GB tumor as a consequence of the two WGD events. Chr7 gain is one of the most commonly reported alterations in GB [34, 45–47]. Furthermore, we additionally detected gains on chromosomes 19 and 20, that are also commonly reported alterations in GB [34, 48]. A previous case study of PLNTY progressing into a higher grade tumor also reported a similar copy number profile, with both primary and recurrent tumors displaying wide loss of heterozygosity with additional gains on chromosomes 7 and 20 [10]. This suggests that the genome-wide losses in chromosomal heterozygosity can pose a risk for malignant transformation of this typically low-grade tumorous lesion. It is important to evaluate the precise risk and incorporate this aspect in the clinical decision-making after gathering additional data about similar cases. Wide chromosomal alterations encompassing whole chromosomes have been reported previously in multiple PLNTY cases [3, 5, 6] suggesting aneuploidy in this tumor type. Currently, this nearly genome-wide loss of heterogeneity has been reported previously only once in PLNTY, and this case also progressed to a more malignant tumor type [10].

Interestingly, BRAF_V600E_ was identified as the driving oncogenic point mutation in our study, whereas Bale et al. [10] reported *FGFR3::TACC3* alteration as an oncogenic driver mutation for both primary PLNTY and recurrent high-grade tumors in their case study. We detected highly positive immunohistochemical stain for BRAF_V600E_ in our E-GB sample but not in the PLNTY-like tumor. PLNTY tumor was highly calcified and went through a routine decalcification process prior to any of our analyses, which most likely has affected the stain of BRAF_V600E_ of the tumor tissue [49]. In addition, *BRAF* is located on chromosome 7, which in our case study had gained additional copies in E-GB, suggesting higher expression of BRAF_V600E_ also at the protein level. Furthermore, in all of the E-GB samples, but not in the PLNTY-like tumor, we detected a homozygous deletion of tumor suppressor *CDKN2A/B* locus, highlighting the critical role of *CDKN2A* inactivation in malignant transformation. Homozygous deletion of 9p21.3 loci, which includes *CDKN2A* is known to be one of the most common alterations in E-GB [13, 14]. Multiple studies have reported the progression of lower grade PXA tumor into a high grade E-GB [13, 20–22] and some studies suggest the role of BRAF_V600E_ to occur as an early event followed by additional driving alterations such as TERT promoter mutation and homozygous deletion of *CDKN2A*, that drive the progression of the PXA into E-GB [50, 51]. Interestingly, to our knowledge, this is the first report of the BRAF_V600E_ mutated PLNTY-like tumor progressing to E-GB. This study together with previous studies will further validate the BRAF_V600E_ accompanied with *CDKN2A* inactivation as one of the possible driving mechanisms for low grade tumor progression into high grade E-GB.

In all E-GB samples, we detected a Q521K point mutation in *EZH2*, which participates in histone modification and gene silencing, as well as in cell proliferation, invasion, and differentiation [52, 53]. EZH2 is commonly overexpressed in epithelioid GB [15–17], and because it is also located on chromosome 7, *EZH2* has gained additional copies in our case study. Q521 is located in the CXC domain (alias pre-SET region) of *EZH2*, which is located just upstream of the SET activation domain. It is challenging to predict the outcome of Q521 mutation, as typically the CXC domain is considered important for functional activity but it has been also reported to potentially contribute to the inactive form of *EZH2* [54, 55]. Another interesting point mutation detected in our analysis was inactivating frameshift deletion of *RBBP6*, which is known to be dysregulated in multiple different tumor types, as well as interacting with common tumor-associated genes RB and p53 [56, 57]. Absence of RBBP6 has also been shown to slow down DNA replication and to contribute to genome instability [58]. Although we consider these genes and alterations interesting, it is unclear whether they contribute to the malignant transformation.

Our analysis detected a high number of point mutations in both FFPE tumor samples. Some of these variants were identified as artifacts likely caused by FFPE sample processing and by the decalcification of the PLNTY-like tumor FFPE sample. Most of the variants in the FFPE samples had low allele fractions, and therefore not all can be considered reliable. Our analysis has focused on variants detected in all tissue samples, including FF tissue and CL, which creates higher confidence for the reported point mutations.

Currently, the recommended treatment option for PLNTY patients is surgical resection, especially if the clinical presentation of the patient is complicated by epilepsy. In many reported cases, surgical resection improves the quality of life and provides seizure-free life for the patient [1, 3, 4, 6, 59]. However, recurrence of epileptic seizures has been reported in some cases [1, 3, 59, 60]. Furthermore, CD34 positivity and BRAF_V600E_ in low-grade epilepsy-associated tumors (LEATs) have been linked to chronic epilepsy [6, 8, 9]. In light of our results, supported by a previous report on malignant transformation of PLNTY [10], surgical resection and molecular genomic characterization of this low-grade tumor could be recommended, not only to treat the epilepsy, but also to manage the risk for possible progression of the tumor to more malignant glioma. We recommend proper molecular evaluation of oncogenic BRAF_V600E_ also for low-grade tumors, because more prevalent treatment resistance to chemotherapy has been observed for BRAF_V600E_ positive tumors, and targeted treatment for BRAF_V600E_ tumors has shown to significantly increase the progression-free survival of the patients [61].

To conclude, we showed here an atypical route to E-GB development from a tumor consistent with PLNTY based on the histological and molecular analysis. Our findings suggest that extensive chromosomal LOH accompanied by oncogenic BRAF_V600E_ can increase the likelihood of a low-grade PLNTY-like lesion progression to a more malignant E-GB. We expect that the homozygous deletion of *CDKN2A* is the main driver of PLNTY-like tumor progression into a PXA-like E-GB, although the whole-genome duplication also took place during this process. Because immunohistochemical analysis of highly calcified PLNTY tumors is challenging, molecular and genomic profiling might serve as an important tool in the diagnosis of PLNTYs and also other low-grade neuroepithelial lesions. Genomic profiling will also give valuable information for the treatment of these tumors, because it might also provide candidate alterations for a targeted therapy.

## Supplementary Information

Additional file 1: Supplementary Figures

Additional file 2: Table S1. Antibodies, incubation times and other reagents used to conduct the immunohistochemical stains for tumor samples.

Additional file 3: Table S2. Karyotyping results for the E-GB patient cell line.

Additional file 4: Table S3. Point mutations detected in the coding regions of the genes in both PLNTY-like tumor and three E-GB samples or in all three E-GB samples, but not in PLNTY-like tumor. The table presents information of the variant location, altered nucleotide and amino acid, functionality, and type of exonic mutation (non-synonymous, synonymous/ frameshift deletion). Number of altered DNA reads, total number of DNA reads and alteration fraction are also reported in all samples (PLNTY-FFPE, E-GB FFPE, E-GB FF and E-GB CL). Number of altered RNA reads, total number of RNA reads and alteration fraction is also reported in E-GB FF and E-GB CL samples. Additionally, the table provides information on whether variant is reported in ClinVar database, Cosmic database, allele frequencies in different populations and prediction scores for variant to be harmful in different prediction algorithms.

Additional file 5: Table S4. Structural variants detected in both PLNTY-like tumor and three E-GB samples or in all three E-GB samples, but not in PLNTY. Table presents information of the variant location, variant type, gene that variants locate to and quality (variants with QUAL<30 were filtered out). Genotype, number of altered DNA reads (AlleleDepth), total number of DNA reads (Depth), genotype quality (GenotypeQual), likelihood of variant to be real (NormalizedLikelihood, LogOddsREF & LOgOddsReal), spanning reads (NSpanningReads) and discordant read pairs supporting the variant (NDiscordantReads) are also reported in all samples (Germline, E-GB FFPE, E-GB FF, E-GB CL and PLNTY).

Additional file 6: Table S5. Germline variants that were shared between the mother and the patient and located in the coding region of the genes in the chromosomes that were originally inherited from the mother and present in the tumor samples. Additionally, genes were filtered based on population frequency 0.1 as well as Cosmic (>1 reported hits) and Clinvar databases and algorithmically predicted pathogenicity. The table presents information of the variant location, altered nucleotide and amino acid, functionality, and type of exonic mutation (non-synonymous, synonymous/ frameshift deletion). Number of altered RNA reads, total number of RNA reads and alteration fraction is reported in E-GB FF and E-GB CL samples. Additionally, the table provides information on whether variant is reported in ClinVar database, Cosmic database, allele frequencies in different populations and prediction scores for variant to be harmful in different prediction algorithms.

## Data Availability

The datasets generated and/or analysed during the current study will be available in the Finnish Federated European Genome-phenome Archive (FEGA) repository once this has been approved based on the legal assessment and all the needed agreements have been made. The access to the data can be requested from the Research Services (Tutkimuspalvelut) of the data controller, the Wellbeing Services County of Pirkanmaa.

## Abbreviations

PLNTY: Polymorphous low grade neuroepithelial of young
E-GB: Epithelioid glioblastoma
IDH: Isocitrate dehydrogenase
GB: Glioblastoma
BRAF_V600E_: BRAF V600E nonsynonymous point mutation
FGFR2: Fibroblast growth factor receptor 2
FGFR3: Fibroblast growth factor receptor 3
MAPK: Mitogen activated kinase
OLIG2: Oligodendrocyte transcription factor 2
NeuN: Neuronal nuclear protein
GFAP: Glial fibrillary acidic protein
TERT: Telomerase reverse transcriptase
MGMT: O-6-methylguanine-DNA methyltransferase
CDKN2A/B: Cyclin dependent kinase inhibitor
EZH2: Enhancer of zeste 2 polycomb repressive complex 2
PXA: Pleomorphic xanthoastrocytomas
WHO: World Health Organization
FFPE: Formalin fixed paraffin embedded
FF: Freshly frozen tissue
CL: Patient derived cell line
NSC: Neural stem cell
WGS: Whole genome sequencing
CNA: Copy number alteration
SV: Structural variation
SNP: Single nucleotide polymorphisms
MRI: Magnetic resonance imaging
CNS: Central nervous system
CN-LOH: Copy-neutral loss of heterozygosity
WGD: Whole genome duplication
SNV: Single nucleotide variants
FOXRED2: FAD dependent oxidoreductase domain containing 2
SSTR5: Somatostatin receptor 5
GNS: Glucosamine (N-acetyl)-6-sulfatase
DCAF8L1: DDB1 and CUL4 associated factor 8 like 1
HS6ST2: Heparan sulfate 6-O-sulfotransferase 2
RASGRF1: Ras protein specific guanine nucleotide releasing factor 1
RBBP6: RB binding protein 6 ubiquitin ligase
PTGER4: Prostaglandin E receptor 4
ACTRT1: Actin related protein T1
HABP2: Hyaluronan binding protein 2
SLC26A1: Solute carrier family 26 member 1
AT/RT: Atypical teratoid/rhabdoid tumor
LEATs: Low-grade epilepsy associated tumors
