# Supplementary material for: Genomic analysis of PLNTY-like tumor progression into epithelioid glioblastoma: a case report
Source: Acta Neuropathol Commun. 2026 Jan 8;14:35. doi: 10.1186/s40478-025-02209-3 (PMC12874860; doi:10.1186/s40478-025-02209-3)
Supplement: Supplementary file 1 — Additional file 1: Supplementary Figures [file 40478_2025_2209_MOESM1_ESM.pdf]

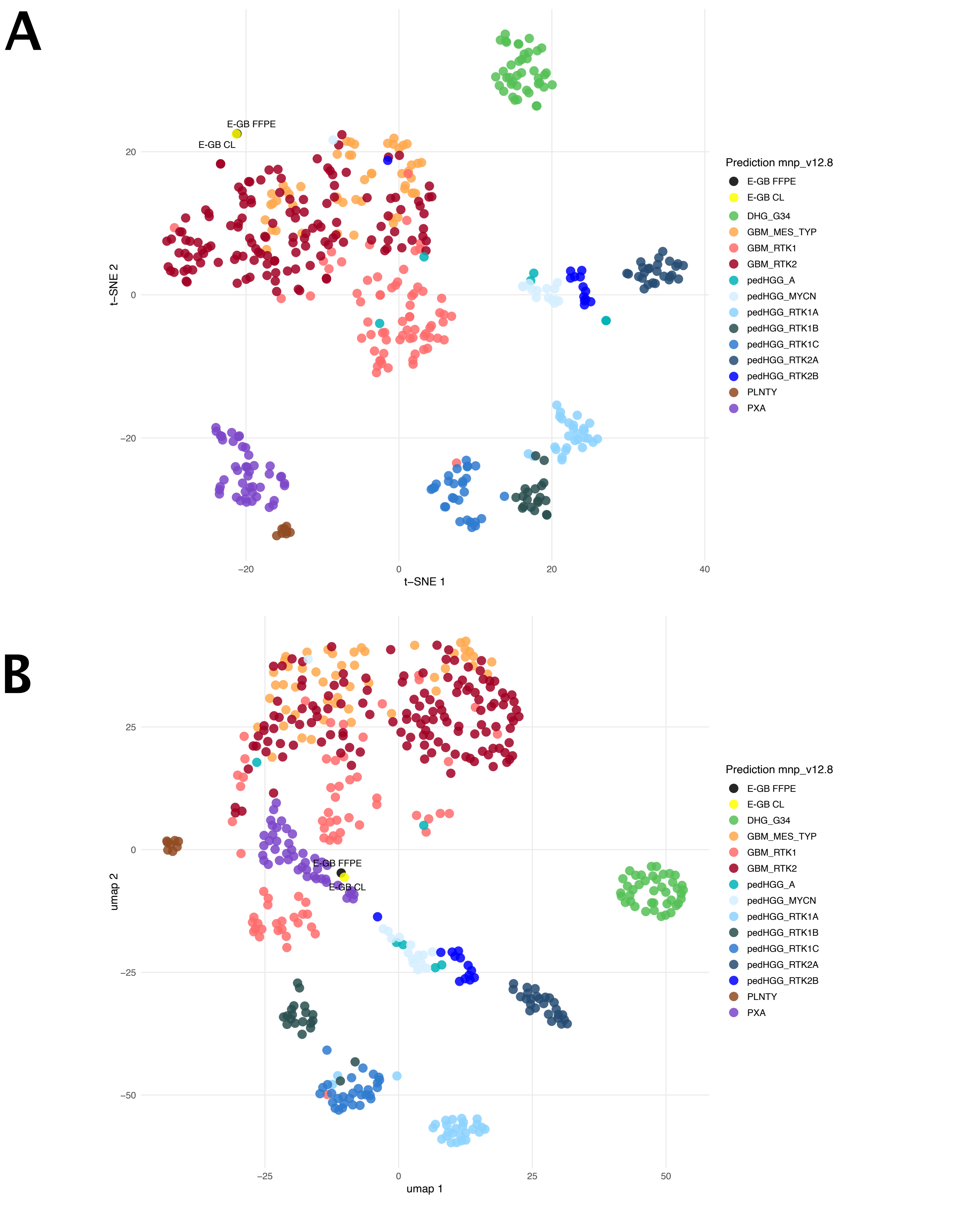

Supplementary Figure 1 | DNA methylation analysis of E-GB (FFPE and CL) (updated model (v12.8.)) classified both samples into subclass (anaplastic) pleomorphic xanthoastrocytoma (PXA) (confidence score 0.98 and 0.63, respectively), positioning the E-GB tumor into a PXA-like E-GB subclass. A) t-SNE presentation of E-GB FFPE and E-GB Cl sample methylation profiling in respect to reference samples B) UMAP presentation of E-GB FFPE and E-GB Cl sample methylation profiling in respect to reference samples.

PLNTY
FFPE
cfrac: 33.5%
avg ploidy: 2.47

GBM
Frozen
cfrac: 77%
avg ploidy: 4.49

GBM
FFPE
cfrac: 73.5%
avg ploidy: 4.65

GBM
Cell line
cfrac: 100%
avg ploidy: 4.17

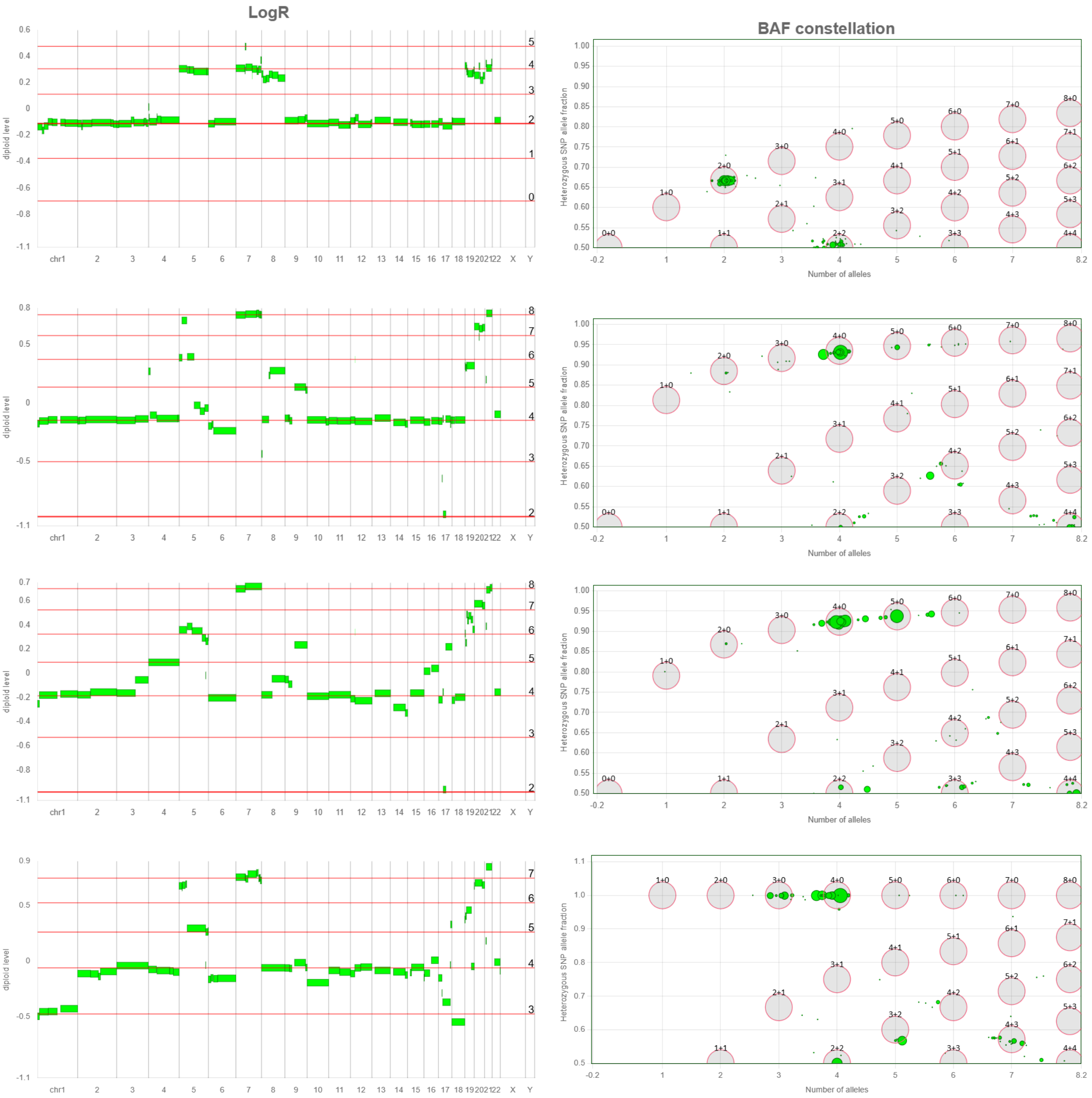

Supplementary Figure 2 | Sample copy number segmentation was analyzed using ASCAT/Battenberg, and the final fitting of the copy number profiles was done using CNsolver (Anssi Nurminen, manuscript under preparation). For each sample, a LogR plot is used to show the number of total copies (red lines) for each detected continuous segment (green bars) in each autosomal chromosome. The BAF constellation plots combine heterozygous SNP allele fraction information inside the segments with the total copy number information to indicate which segments are detected as clonal (landing inside the grey circles) in the sampled cancer cell populations. cfrac indicates the estimated cancer cell fraction, avg ploidy the average ploidy of the cancer cells.

A

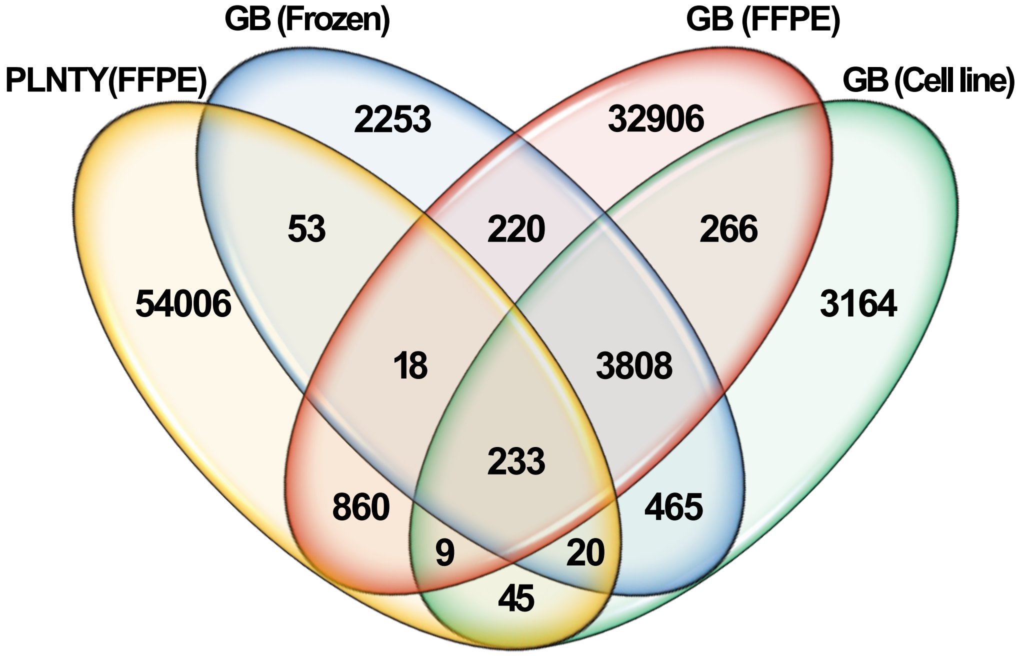

B

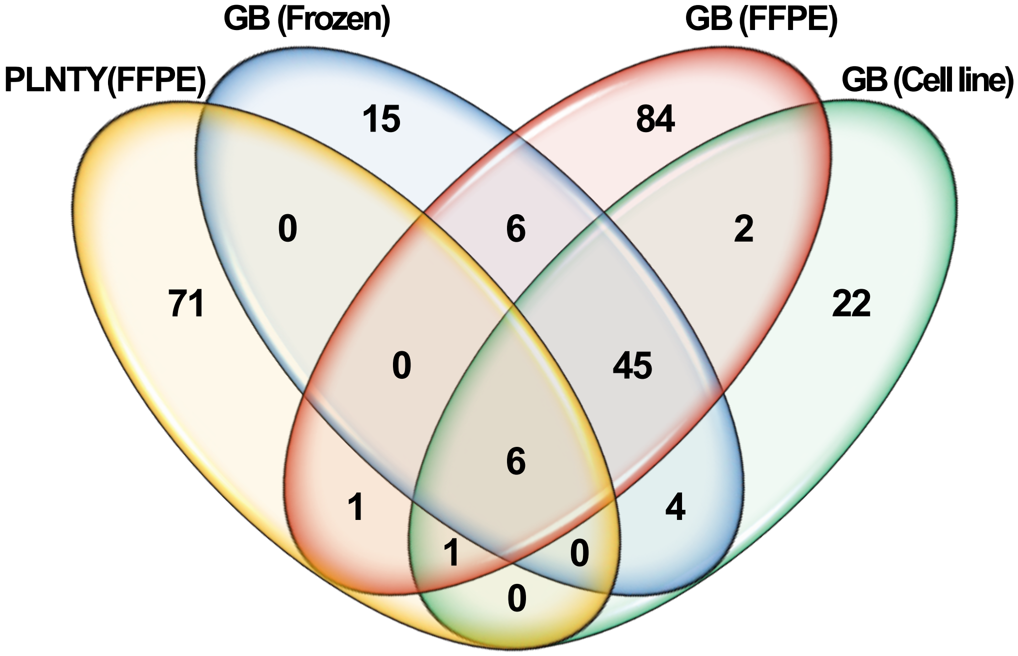

Supplementary Figure 3 | A) Venn diagram presenting the number of shared somatic point mutations between PLNTY-like tumor (FFPE) and the three E-GB samples (FFPE, FF and CL). Altogether 233 mutations were shared between all four tumor samples. PLNTY-like tumor (FFPE) and E-GB FFPE samples seemed to carry a lot of unique point mutations, which are likely at least partly technical artifacts related to sample handling. B) Venn diagram presentation of somatic coding mutations revealed six nonsynonymous coding or exonic long noncoding RNA mutations shared between PLNTY-like tumor and all E-GB samples. In addition to nonsynonymous mutations in BRAF, GNS, FOXRED2, and SSTR5 genes, exonic variants in two long non-coding RNAs, LINC02417 (chr12:3371539, T>C) and LINC01684 (chr21:24547081, T>C) were identified in both PLNTY-like tumor and all the E-GB tumors. All three E-GB samples (FFPE, FF and CL) shared 45 nonsynonymous coding or exonic long noncoding RNA mutations that were not detected in PLNTY-like tumor.

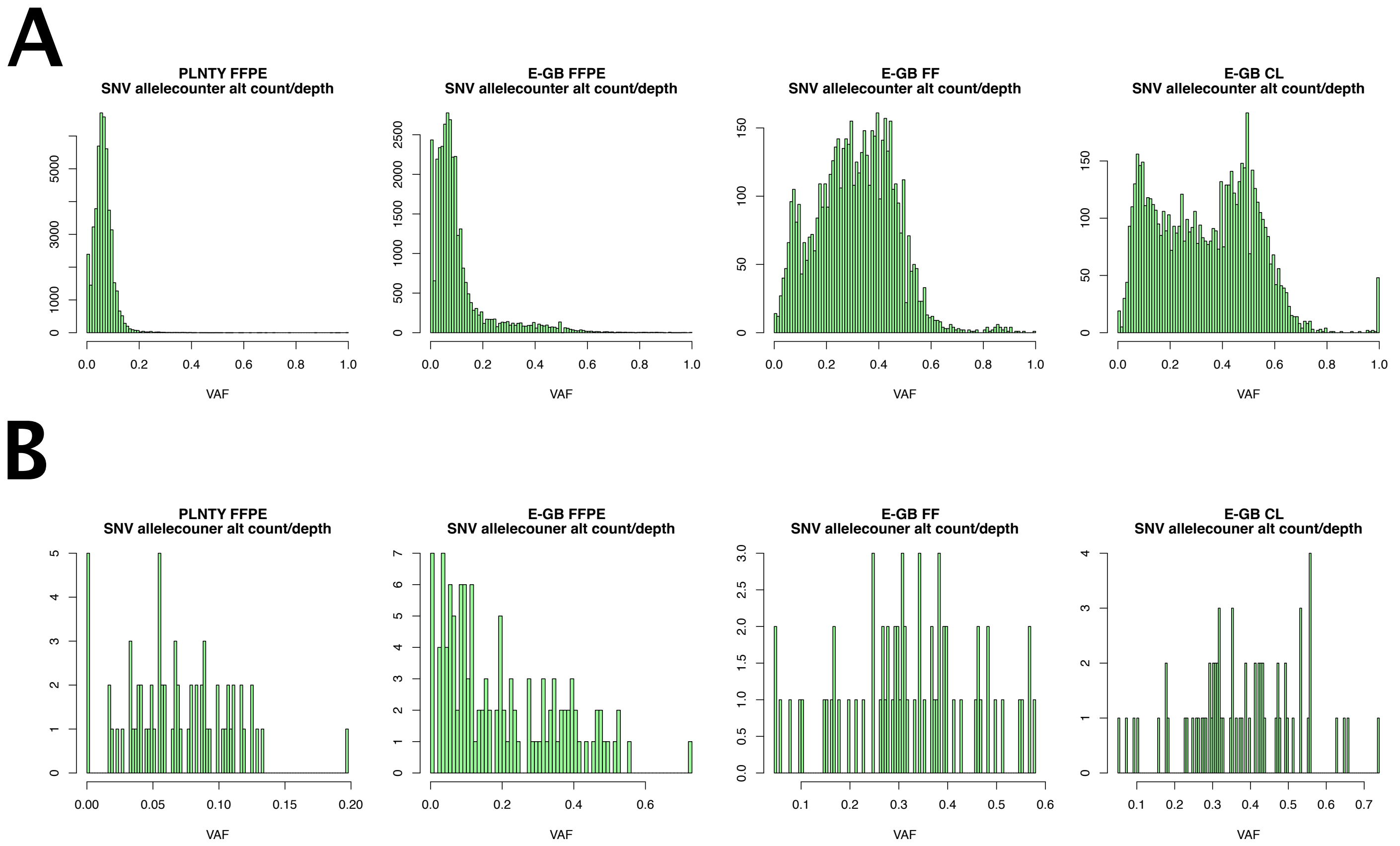

Supplementary Figure 4 | Alternative allele fraction distributions in A) all sample SNVs and B) SNVs affecting exonic regions show that FFPE samples have a high amount of variants with low allele fractions compared to FF and CL samples.

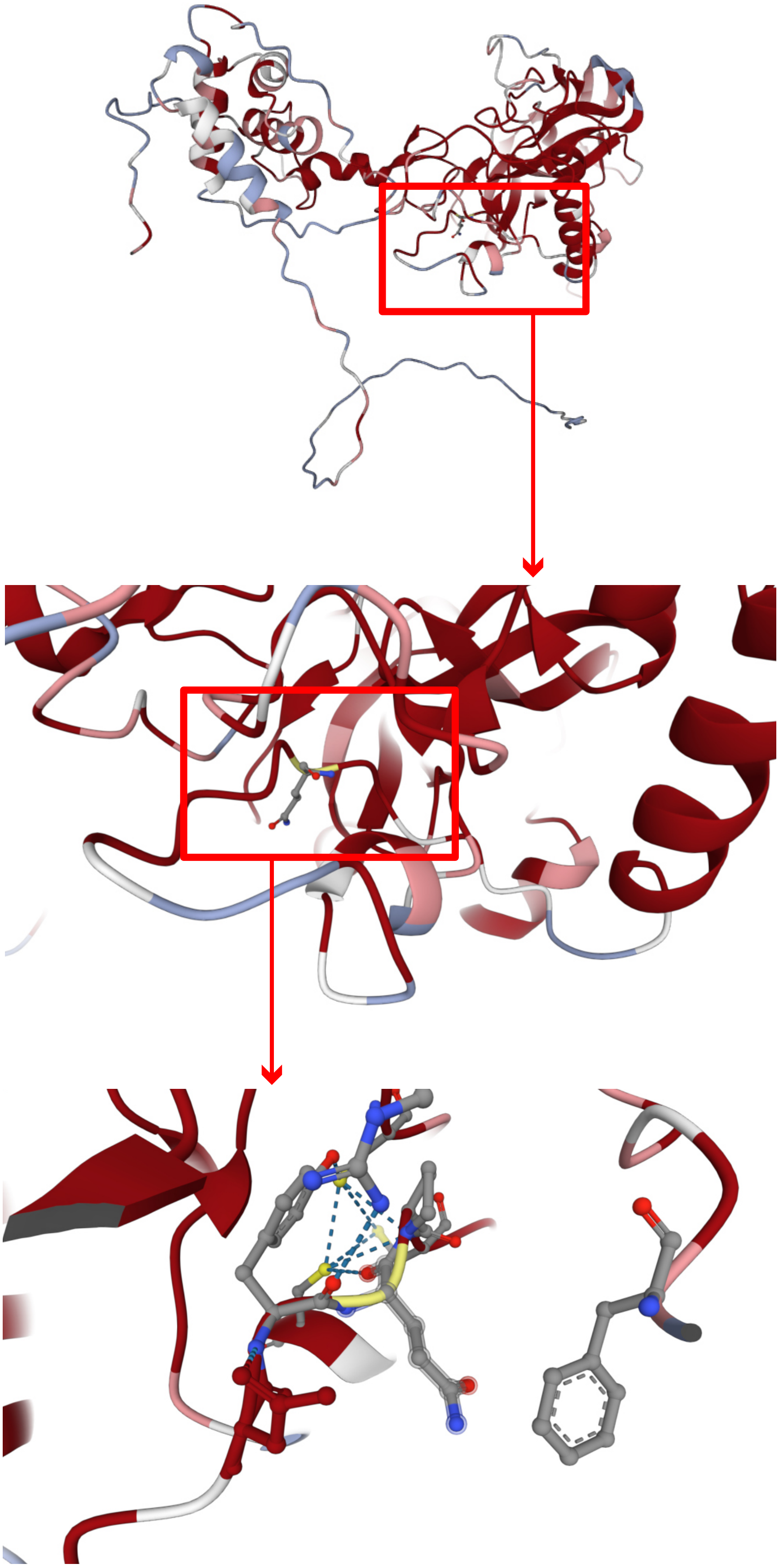

Supplementary Figure 5 | In all E-GB samples (FFPE, FF and CL) a non-synonymous SNV in EZH2 was detected. The CXC domain of the gene had a substitution of C>A, which leads to glutamine substitution to lysine in the EZH2 protein, changing the negative charge to a positive charge in the respective position (Chr7, exon14:c.C1561A:p.Q521K in transcript NM_001203247).
